# Targeting glucocorticoid receptors prevents the effects of early life stress on amyloid pathology and cognitive performance in APP/PS1 mice

**DOI:** 10.1038/s41398-018-0101-2

**Published:** 2018-03-01

**Authors:** Sylvie L Lesuis, Sascha Weggen, Sandra Baches, Paul J Lucassen, Harm J Krugers

**Affiliations:** 1 Swammerdam Institute for Life Sciences, Centre for Neuroscience, University of Amsterdam, Science Park 904, 1098 XH Amsterdam, The Netherlands; 2 Department of Neuropathology, Heinrich-Heine-University, Moorenstrasse 5, D-40225 Düsseldorf, Germany

## Abstract

Exposure to chronic stress or elevated glucocorticoid hormone levels in adult life has been associated with cognitive deficits and an increased risk for Alzheimer’s disease (AD). Since exposure to stress during early life enhances stress-responsiveness and lastingly affects cognition in adult life, we here investigated; (i) whether chronic early life stress (ELS) affects AD pathology and cognition in middle-aged APPswe/PS1dE9 mice, and (ii) whether it is still possible to rescue these late effects by briefly blocking glucocorticoid receptors (GRs) at a translationally relevant, middle age. Transgenic APPswe/PS1dE9 mice were subjected to ELS by housing dams and pups with limited nesting and bedding material from postnatal days 2–9 only. In 6- and 12-month-old offspring, this resulted in enhanced hippocampal amyloid-β (Aβ)-40 and -42 levels, and in reduced cognitive flexibility, that correlated well with the Aβ42 levels. In parallel, CORT levels and BACE1 levels were significantly elevated. Surprisingly, blocking GRs for only 3 days at 12 months of age reduced CORT levels, reduced hippocampal Aβ40 and -42, and β-site APP-cleaving enzyme 1 (BACE1) levels, and notably rescued the cognitive deficits in 12-month-old APPswe/PS1dE9 mice. These mouse data demonstrate that exposure to stress during the sensitive period early in life influences later amyloid pathology and cognition in genetically predisposed, mutant mice, and as such, may increase AD vulnerability. The fact that a short treatment with a GR antagonist at middle age lastingly reduced Aβ levels and rescued the cognitive deficits after ELS, highlights the therapeutic potential of this drug for reducing amyloid pathology.

## Introduction

The mechanisms that underlie sporadic Alzheimer’s disease (AD) remain largely elusive, which hampers the development of successful intervention strategies for AD. While familial forms of AD can be explained by genetic causes—often related to changes in amyloid-β (Aβ) (e.g. refs ^1,2^)—sporadic AD likely has a multifactorial aetiology, in which, next to amyloid, also lifestyle factors play an important role^2–5^.

Stress is an important environmental risk factor that has been implicated in AD progression^6,7^. Clinical observations suggest that stressful life events can reduce the age of onset in AD^6^, while stress-related disorders like depression can promote AD symptoms and neuropathology^8^. Glucocorticoid hormones (GCs; cortisol in humans and corticosterone (CORT) in rodents) are powerful steroids released in response to stress. They are often increased in AD, notably already in early stages of the disease^9,10^, and dysregulation of the hypothalamus–pituitary–adrenal (HPA) axis is also associated with a higher AD risk^8,10,11^. Rodent studies further demonstrate that exposure to stress and/or elevated GC levels at an adult age impairs cognition and enhances Aβ levels both in mutant^12–16^ and in wild type (WT) animals^17,18^.

The early postnatal period is a particularly sensitive time window that determines sensitivity to stress and cognitive function in later life. As exposure to early life stress (ELS) in WT mice is well-known to accelerate cognitive decline^19,20^, we tested the hypothesis that ELS—induced by housing mice with limited nesting and bedding material from postnatal days (PND) 2–9^21–24^—increases the development of AD pathology and cognitive decline in APPswe/PS1dE9 mice, a classic mouse model for amyloid pathology^25^. Secondly, in order to study whether glucocorticoids are instrumental, we tested whether briefly targeting glucocorticoid receptors (GRs) could rescue late effects of ELS on AD-related pathology and cognitive performance. We therefore treated animals with mifepristone, which is a Food and Drug Administration-approved drug that selectively blocks GRs at high concentrations and is prescribed to treat Cushing’s disease. It has further been tested in preliminary studies on (aspects of) AD^26^ and psychotic depression^27,28^ (see ref. ^29^ for a review about the function and applicability of mifepristone in humans).

## Materials and methods

### Mice and breeding

In this study, we conducted experiments under Dutch national law as well as under European Union directives on animal experiments. The animal welfare committee of the University of Amsterdam approved all experiments. Mice were housed at a temperature of 20–22 °C. Humidity was between 40 and 60%, and animals were fed ad libitum with standard chow and water. Lights were on between 8.00 a.m. and 8.00 p.m. unless stated otherwise. WT and APPswe/PS1dE9^25^ male littermates of 6 and 12 (±1) months of age were used. To obtain mice, two 10-week-old C57Bl/6J virgin WT females (Harlan Laboratories B.V., Venray, The Netherlands) and one heterozygous male APPswe/PS1dE9 mouse were housed together for 1 week to allow mating. Individually housed pregnant females were monitored daily for the birth of pups. For litters born before 10.00 a.m., the day of birth (PND 0) was considered the previous day, after which the ELS paradigm was initiated from PND 2 to 9. At PND 21, mice were weaned and ear tissue was collected for identification and genotyping. Littermates were housed with 2–6 mice per cage. All animals were undisturbed (except for cage cleaning once a week) until the start of the experimental procedures. Number of mice—6 months: Ctrl-WT, 12; Ctrl-APPswe/PS1dE9, 10; ELS-WT, 11; and ELS-APPswe/PS1dE9, 14; 12 months: Ctrl-WT, 16; Ctrl-APPswe/PS1dE9, 11; ELS-WT, 19; and ELS-APPswe/PS1dE9, 12. Group sizes were chosen to ensure sufficient statistical power.

### Early life stress

At PND 2, litters were culled to six pups per litter, and dams and their litters were randomly assigned to the ELS or control (Ctrl) condition until PND 9, after which all mice were treated equally, as described before^21–24^. Briefly, the Ctrl condition consisted of cages with standard amounts of nesting and bedding material (one piece of nesting material (5x5 cm; Technilab-BMI, Someren, the Netherlands)). In the ELS condition, a fine-gauge stainless steel mesh was placed in the cage, with a small amount of sawdust bedding and ½ piece of nesting material.

### Maternal behaviour

Maternal behaviour was observed daily from PND 3 until PND 8 at 9.00 a.m. and 8.30 p.m as described previously^30^. Briefly, the level of activity of the dam was scored in sixteen 1-min epochs per day, spread over 48 min observation sessions. The behaviours that were scored were: licking and grooming behaviour; nursing behaviour; and the time that the dam spent off the pups.

### Barnes maze

Six- and twelve-month-old APPswe/PS1dE9 and WT male mice were tested for spatial memory in the spatial Barnes maze task. Testing occurred during the dark, active phase in the afternoon (1 p.m.). A classic setup was used (110 cm diameter, 12 exit holes) in which mice were placed in the centre of the maze twice daily (inter-trial interval of 30 min) for 4 consecutive days and allowed to navigate to the exit hole leading to the home cage (acquisition learning). A probe trial (all holes closed) was conducted 24 h after the last trial. Following the probe trial, behavioural flexibility was tested by relocating the exit hole to another location on the maze (150°) for 4 days (reversal learning), followed by a probe trial. Cages containing used bedding material were placed at equal distance under the maze to avoid guidance by odour cues, the board was rotated after each trial and the maze was cleaned with 25 % EtOH to dissipate odour cues. Distal extra-maze cues were always fixed relative to the exit hole. Performance of the mouse was assessed by an observer blind to the experimental condition of the mouse.

### Stress response

Two acute stressors were used to determine stress responsiveness; a 6-min forced swim test (original experiment) or an acute 0.4 mA foot shock (mifepristone experiment). Blood samples were collected by a tail cut at 30 min (peak stress response) and 90 min (stress recovery) after the stressor. A commercially available radioimmunoassay kit (MP Biomedicals, Eindhoven, The Netherlands) was used to measure plasma CORT levels.

### Tissue preparation

Following behavioural testing, mice were sacrificed by quick decapitation, between 8.00 and 9.00 p.m. (beginning of the inactive phase), i.e., when plasma CORT levels are low. Blood plasma was collected and CORT levels were determined as described above.

### Diaminobenzidine immunohistochemistry

For diaminobenzidine (DAB) immunohistochemistry, pre-mounted sections (40 µm) on glass slides (Superfrost Plus slides, Menzel) were dried overnight. Endogenous peroxidase activity was blocked using 0.3% H_2_O_2_ for 15 min, after which sections were boiled in a microwave (±95 °C) in citrate buffer (0.01 M, pH 6.0, 15 min). To block non-specific staining, slices were incubated for 1 h in blocking mix (0.05 M Tris-buffered saline (TBS) containing 1% bovine albumin serum (BSA) and 0.1% triton), and primary antibody mix (6E10 (1:1500, Bioline) in blocking mix) was applied for 2 h at room temperature and overnight at 4 °C. Secondary antibody (1:200, sheep anti-mouse biotinylated (GE Healtcare)) was applied for 2 h, after which sections were treated with avidin-biotin complex (ABC, 1:800, Vectastain elite ABC-peroxidase kit, Brunschwig Chemie). Chromagen development was conducted by incubation in 0.05 M Tris buffer containing 0.01% H_2_O_2_ and 0.2 mg/ml DAB.

### Imaging and quantification

Plaque load was quantified for all APPswe/PS1dE9 mice by an experimenter who was blind to the experimental conditions. Using a Nikon DS-Ri2 microscope, representative images (×10 magnification) were captured and analysed using ImageJ. A fixed intensity threshold was applied to 8-bit binarised pictures to define the DAB staining. The percentage of area covered by DAB staining was analysed as described previously^31^.

### Western blotting

Following quick decapitation and dissection, hippocampi were snap-frozen and homogenised in RIPA buffer (150 mM NaCl, 1% NP-40, 0.5% sodium deoxycholate, 0.1% SDS, pH = 6.8). Homogenates were sonicated for 2 × 30 s (max intensity), and centrifuged for 1 min (10 000 × *g*, 4°C). Protein concentration was determined in the supernatant by BCA Protein Assay (Pierce, The Netherlands). 15 µg protein was separated on 12.5% polyacrylamide-SDS gels using electrophoresis, and proteins were transferred to polyvinylidene difluoride membranes at 75 V in Towbin buffer (25 mM Tris, 192 mM glycine, 20% methanol, pH = 8.3). Five per cent BSA in TBST (0.1 M TBS + 0.1% Tween-20) was used to block the membranes, and membranes were incubated overnight at 4 °C with primary antibody. Proteins studied were as follows: amyloid precursor protein (APP; 6E10, 1:1500, BioLegend, 100 kDa); β-site APP-cleaving enzyme 1 (BACE1; D10E5, 1:1000, Cell Signalling, 70 kDa); actin (A2066, Sigma-Aldrich, 42 kDa); and GAPDH (14C10, 1:3000, Cell Signalling, 37 kDa). Secondary antibodies were incubated for 2 h, and signal was developed (Licor Odyssey FC; Leusden, the Netherlands). Using ImageJ (NIH; Bethesda) signal intensities were measured and normalised against GAPDH or actin as internal marker. Three independent replications were made, and protein levels were analysed as the mean of these replicates, and expressed as % of Ctrl-APPswe/PS1dE9 levels.

### SDS-soluble Aβ levels

Aβ40 and -42 peptide levels were determined in whole hippocampal homogenates using a sandwich enzyme-linked immunosorbent assay (ELISA) as described previously^32^.

### Mifepristone treatment

Mifepristone (Sigma; 40 mg/ml dissolved in 99.9% EtOH and diluted 20× in arachide oil) was injected intraperitoneally for 3 consecutive days (final dose: 10 mg/kg; injection volume: 5 µl/g body weight) between PND 339–341 (±7 days)^23^. The appropriate vehicle solution was administered accordingly.

### Statistical analysis

Data were analysed using SPSS 22.0 (IBM software). Data are expressed as mean ± standard error of the mean. Data were considered statistically significant when *p* < 0.05 (two-sided testing). Animals/observations were excluded in case of technical failures, following spontaneous death and/or if identified as a significant outlier using a Grubb’s test. Unpaired Student’s *t*-tests (or non-parametric equivalent) were performed to assess differences between two groups. To compare between groups accounting for the main and interaction effects of genotype (WT vs. APPswe/PS1dE9) and condition (Ctrl vs. ELS), a two-way analysis of variance (ANOVA) was performed, with a Tukey post hoc test. A repeated measures ANOVA was performed to assess Barnes maze learning curves over the different trials, and to measure differences in plasma CORT response values. Greenhouse-Geisser correction was applied when the assumption of sphericity was violated. One-sample *t*-test was used to compare performance on the probe trials of the Barnes maze against chance level (25%). Pearson’s correlation test was conducted to determine correlations.

## Results

### ELS results in fragmented maternal care for the offspring

We subjected APPswe/PS1dE9^25^ and WT mice to the well-characterised paradigm of housing with limited nesting and bedding material from PND 2 to 9. This resulted in fragmented maternal care, as indicated by increased exits of the dam from the nest, increased numbers of pups outside the nest and a reduced body weight gain of the pups between PND 2 to 9, consistent with previous reports^21–24^ (Supplementary Table 1). Since the effects of ELS are particularly sex-specific^24^, all experiments were further conducted with male mice.

### ELS increases amyloid pathology

**Fig. 1:**
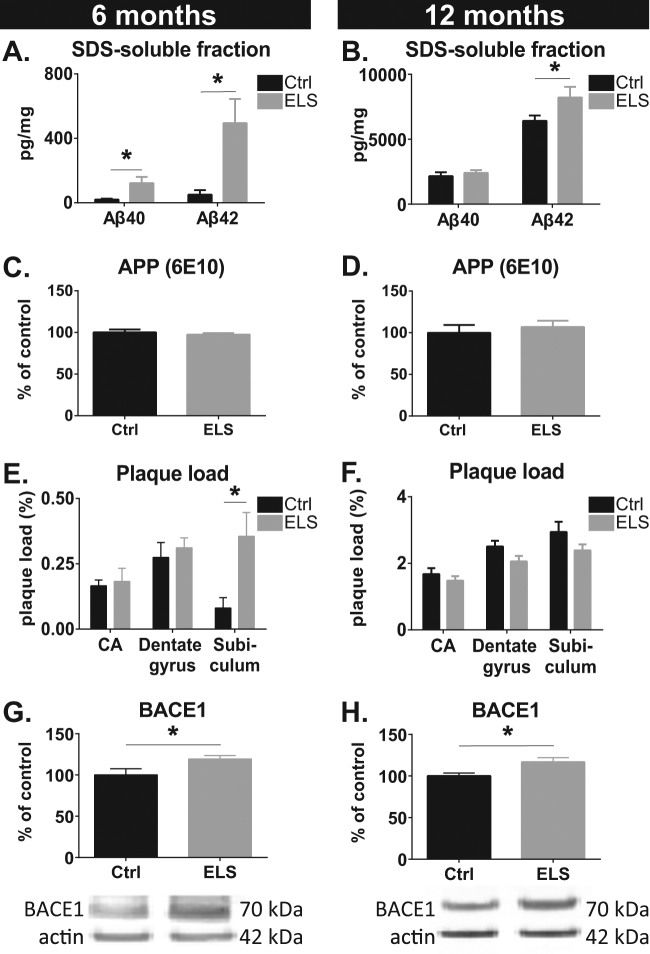
Hippocampal amyloid pathology in male APPswe/PS1dE9 mice. **a** The levels of SDS-soluble Aβ40 and Aβ42 are elevated as a consequence of ELS at 6 months. **b** At 12 months of age, ELS led to an elevation in Aβ42 levels, but not in Aβ40 levels. **c**, **d** No difference was observed between Ctrl and ELS mice for full-length APP levels as measured with 6E10 antibody at 100 kDa at 6 months (**c**) and 12 months (**d**). (**e**) ELS significantly increased amyloid plaque load as measured with 6E10 antibody in the subiculum, but not in the dentate gyrus and cornu ammonis (CA) 1, 2 and 3 areas at 6 months. **f** ELS did not affect plaque load in the subiculum, dentate gyrus or CA areas at 12 months. **g**, **h** BACE1 expression was significantly increased in ELS-APPswe/PS1dE9 mice at 6 (**g**) and 12 months (**h**). Six months: Ctrl, *N* = 5 and ELS, *N* = 8–10; 12 months: Ctrl, *N* = 7–9 and ELS, *N* = 8–9 mice/group

ELISA analysis of whole hippocampal homogenates revealed that the levels of both Aβ40 and the more aggregation-prone Aβ42 peptide were strongly elevated in ELS-APPswe/PS1dE9 transgenic mice at 6 months of age (Aβ40: *t*(10.35) = −2.39, *p* = 0.038; Aβ42: *t*(10.74) = −2.74, *p* = 0.02; Fig. 1a). At 12 months of age, when the amyloid plaque pathology had progressed substantially, SDS-soluble Aβ42, but not Aβ40, levels were significantly elevated in ELS-APPswe/PS1dE9 mice (Aβ40: *t*(16) = −0.80, *p* = 0.44; Aβ42: *t*(8.36) = −2.97, *p* = 0.017; Fig. 1b). Protein levels of full-length APP were not different between Ctrl and ELS APPswe/PS1dE9 mice at these ages (6 months: *t*(8) = 0.66, *p* = 0.53; 12 months: *t*(13) = 0.59, *p* = 0.57; Fig. 1c, d).

Stereological quantification revealed that the percentage of area covered by Aβ plaques was significantly increased in the subiculum of 6-month-old ELS-APPswe/PS1dE9 mice (*t*(7) = 2.52, *p* = 0.04) relative to Ctrl-APPswe/PS1dE9 mice, but was not different in the dentate gyrus (DG) (*t*(11) = 0.57, *p* = 0.57) or cornu ammonis (CA) subregions (*t*(9) = 0.19, *p* = 0.85; Fig. 1e). In 12-month-old AD mice, no differences were observed in the subiculum (*t*(15) = 1.60, *p* = 0.13), DG (*t*(15) = 1.89, *p* = 0.08) or CA areas (*t*(16) = 0.88, *p* = 0.39) between Ctrl and ELS conditions (Fig. 1f). As no Aβ peptides, APP or plaques were detected in Ctrl and ELS WT mice (data not shown), these results reveal that ELS lastingly increases hippocampal SDS-soluble Aβ peptide levels in APPswe/PS1dE9 mice, starting from a relatively early age onwards.

As a potential mechanism by which ELS could increase Aβ levels, we examined the expression of BACE1. In 6 and 12 month-old-mice of age, ELS significantly increased BACE1 expression in APPswe/PS1dE9 mice relative to Ctrl-APPswe/PS1dE9 mice (6 months: *t*(13) = 2.46, *p* = 0.03; 12 months: *t*(11) = 2.72, *p* = 0.01; Fig. 1g, h).

### ELS does not alter cognitive performance in 6-month-old APPswe/PS1dE9 mice

**Fig. 2:**
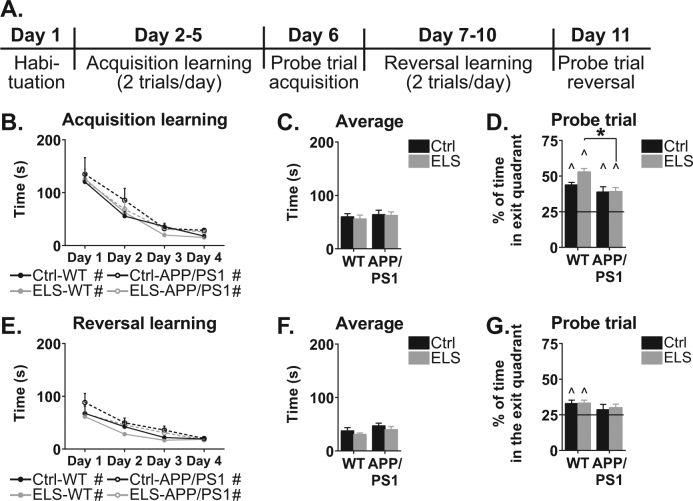
Barnes maze performance of 6-month-old WT and APPswe/PS1dE9 mice. **a** Schematic overview of the experimental design. **b** Acquisition learning was comparable between all four groups. All groups display a significant learning curve over the trials. **c** No difference in the average time to find the exit hole. **d** During the probe trial all groups spend significantly more than 25% of the time in the quadrant where the exit hole was located. ELS-APPswe/PS1dE9 mice spend significantly less time in the exit quadrant than ELS-WT mice. **e** During reversal learning, all groups show comparable latencies to find the exit hole, and all groups had a significant decrease in latency over the days. **f** No differences were observed in the average time to find the exit hole. **g** On the probe trial after reversal learning, only WT mice spend more than 25% of the total time in the exit quadrant. *N* = 8–12 mice/group. * indicates a significant post hoc Tukey test. # indicates a significant learning curve over the days. ^ indicates significant performance compared to chance level

To assess whether the alterations in amyloid levels after ELS were accompanied by behavioural changes, we tested spatial navigation and cognitive flexibility in the Barnes maze (see time line in Fig. 2a). At 6 months of age, mice from all groups were able to locate the exit hole comparably (acquisition learning: condition effect: F(1,38) = 0.02, *p* = 0.90; genotype effect: F(1,38) = 1.03, *p* = 0.32; interaction effect: F(1,38) = 0.02, *p* = 0.88), (average: condition effect: F(1,43) = 0.13, *p* = 0.72; genotype effect: F(1,43) = 0.54, *p* = 0.47; interaction effect: F(1,43) = 0.03, *p* = 0.87; Fig. 2b, c). All mice could identify the exit quadrant in the probe trial, as assessed by significantly more than 25% (chance level) of time spend in the exit quadrant (Ctrl-WT: *t*(10) = 10.51, *p* < 0.001; Ctrl-APPswe/PS1dE9: *t*(6) = 3.69, *p* = 0.01; ELS-WT: *t*(9) = 11.97, *p* < 0.001; ELS-APPswe/PS1dE9: *t*(14) = 5.33, *p* < 0.001; Fig. 2d). However, during the probe trial, ELS-APPswe/PS1dE9 mice performed less well compared to ELS-WT mice (genotype effect: F(1,39) = 11.56, *p* < 0.001; ELS-WT vs. ELS-APPswe/PS1dE9: *p* < 0.001).

After acquisition learning, the same mice were trained in a reversal paradigm, in which the exit hole was relocated 150° (reversal learning). No differences in the time required to locate the exit hole were present between the groups (reversal learning: condition effect: F(1,39) = 1.97, *p* = 0.17; genotype effect: F(1,39) = 3.61, *p* = 0.07; interaction effect: F(1,39) = 0.02, *p* = 0.88), (average: condition effect: F(1,41) = 1.52, *p* = 0.23; genotype effect: F(1,41) = 2.85, *p* = 0.10; interaction effect: F(1,41) = 0.00, *p* = 0.98; Fig. 2e, f), and no between-group effects were present on the probe trial either (condition effect: F(1,40) = 0.12, *p* = 0.74; genotype effect: (F1,40) = 2.03, *p* = 0.16; interaction effect: F(1,40) = 0.04, *p* = 0.84; Fig. 2g).

### ELS hampers reversal learning in 12-month-old APPswe/PS1dE9 mice

**Fig. 3:**
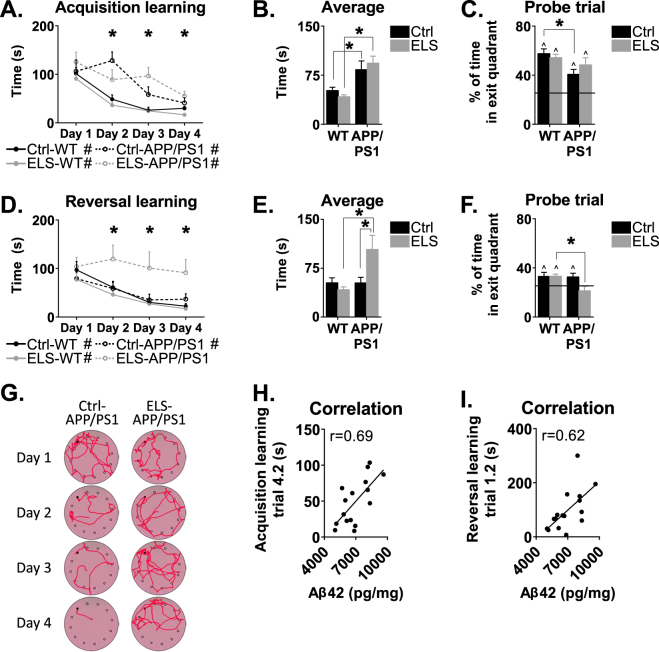
Barnes maze performance of 12-month-old WT and APPswe/PS1dE9 mice. **a** Acquisition learning was significantly slower in APPswe/PS1dE9 mice compared to WT mice on days 2, 3 and 4. All groups showed a significant learning curve over the trials. **b** The average time to locate the exit hole was higher in APPswe/PS1dE9 mice, with significant post hoc differences between Ctrl-WT and Ctrl-APPswe/PS1dE9 mice, and between ELS-WT and ELS-APPswe/PS1dE9 mice. **c** On the probe trial, Ctrl-APPswe/PS1dE9 mice performed significantly worse than Ctrl-WT mice. **d** During reversal learning, there was a significant difference between the groups on day 2, 3 and 4 in the latency to find the exit hole. Overall, all groups showed a significant learning curve, except for ELS-APPswe/PS1dE9 mice. **e** The average time to locate the exit hole was higher in the ELS-APPswe/PS1dE9 mice compared to Ctrl-APPswe/PS1dE9 mice, and compared to ELS-WT mice. **f** On the probe trial, ELS-APPswe/PS1dE9 mice were the only group that did not spend more than 25% of the total time in the exit quadrant. **g** A typical search pattern of APPswe/PS1dE9 mice reared under control (left) or ELS (right) conditions. **h** Performance on the final trial during acquisition learning of the Barnes maze correlated significantly with Aβ42 levels (*r* = 0.69, *p* < 0.05). **i** Performance on the second trial of reversal learning of the Barnes maze correlated significantly with Aβ42 levels in APPswe/PS1dE9 mice (*r* = 0.62, *p* < 0.05). *N* = 8–18 mice/group. * indicates a significant post hoc Tukey test. # indicates a significant learning curve over the days. ^ indicates significant performance compared to chance level

At 12 months of age, APPswe/PS1dE9 mice overall required more time to locate the exit hole when compared to WT type mice (genotype effect: F(1,47) = 37.25, *p* < 0.001), and displayed significantly longer latencies to find the exit hole over training days 2–4 (day 2: F(1,47) = 31.22, *p* < 0.001; day 3: F(1,47) = 27.28, *p* < 0.001; day 4: F(1,47) = 16.33, *p* < 0.001; Fig. 3a). These findings were also reflected in the average time that the mice required to locate the exit hole, which was higher in both groups of APPswe/PS1dE9 mice when compared to WT mice (F(1,47) = 32.72, *p* < 0.001; post hoc: Ctrl-WT vs. Ctrl-APPswe/PS1dE9, *p* = 0.02; ELS-WT vs. ELS-APPswe/PS1dE9, *p* < 0.001; Fig. 3b). During the probe trial, Ctrl-APPswe/PS1dE9 mice spend less time in the exit quadrant than Ctrl-WT mice (genotype effect: F(1,45) = 7.54, *p* = 0.01; post hoc: *p* = 0.04; Fig. 3c). Importantly, no differences were observed between the groups in walking distance or speed during habituation (genotype effect: F(1,46) = 2.66, *p* = 0.11; condition effect: F(1,46) = 2.81, *p* = 0.10), ruling out a priori differences in locomotor activity (data not shown).

During reversal learning, only ELS-APPswe/PS1dE9 mice were unable to locate the exit hole, as indicated by a significant overall difference in reversal learning (interaction effect: F(1,43) = 9.52, *p* = 0.004) and their failure to decrease the time to the exit hole over the trials (Ctrl-WT: F(1.58,18.90) = 12.11, *p* < 0.001; ELS-WT: F(1.54,26.15) = 13.64, *p* < 0.001; Ctrl-APPswe/PS1dE9: F(3,21) = 4.25, *p* = 0.02; ELS-APPswe/PS1dE9: F(3,21) = 0.35, *p* = 0.79; Fig. 3d) and higher average escape latencies across all trials (interaction effect: F(1,43) = 9.52, *p* < 0.001; post hoc test: ELS-WT vs. ELS-APPswe/PS1dE9, *p* < 0.001; Ctrl-APPswe/PS1dE9 vs. ELS-APPswe/PS1dE9, *p* = 0.02; Fig. 3e).

This was confirmed by the probe trial, where ELS-APPswe/PS1dE9 mice spend less time in the exit quadrant than ELS-WT mice (genotype effect: F(1,45) = 4.32, *p* = 0.04; post hoc test: *p* = 0.02), and only the ELS-APPswe/PS1dE9 mice spend <25% of the time (chance level) in the escape quadrant (Fig. 3f). This effect is illustrated by the movement trajectories of the ELS-APPswe/PS1dE9 mice, which showed little improvement in these animals over the trial days compared to Ctrl-APPswe/PS1dE9 mice (Fig. 3g).

Notably, the latency to locate the exit hole on the final trial of the acquisition phase of the Barnes maze (trial 4.2), which is the most reliable trial to assess the extent to which mice have learned to successfully navigate to the exit hole, correlated positively with hippocampal Aβ42 levels in 12-month-old APPswe/PS1dE9 mice (*r* = 0.69, *n* = 16, *p* < 0.001; Fig. 3h). Moreover, hippocampal Aβ42 levels correlated positively with the latency to locate the exit hole on the second trial of the reversal learning phase (trial 1.2; *r* = 0.62, *n* = 16, *p* = 0.01; Fig. 3i), which is the first trial in which the ability of the animal to adapt to the relocated position of the exit hole becomes apparent (i.e., the trial in which the mice need cognitive flexibility). Since the behavioural tests revealed a phenotype only in ELS-APPswe/PS1dE9 mice at 12 months of age, all subsequent experiments were conducted with this age group.

### CORT levels and ELS

**Fig. 4:**
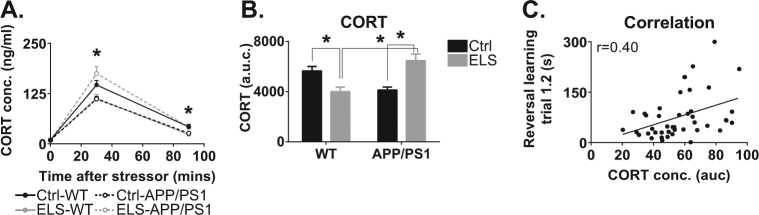
HPA axis activity is affected by early life experiences and a transgenic background at 12 months of age. **a** In 12-month-old mice, ELS-APPswe/PS1dE9 mice have elevated CORT levels 30 and 90 min after a stressor compared to Ctrl-APPswe/PS1dE9 mice and ELS-WT mice. **b** The overall CORT exposure after a stressor is higher in 12-month-old ELS-APPswe/PS1dE9 mice compared to all other groups. ELS-WT mice have lower CORT levels than Ctrl-WT mice and ELS-APPswe/PS1dE9 mice. **c** CORT levels are significantly correlated to performance on trial 1.2 of the reversal learning phase of the Barnes maze (*r* = 0.40, *N* = 44, *p* = 0.01). *N* = 8–18 mice/group. *Significant post hoc Tukey test

To evaluate whether alterations in responsiveness of the HPA axis were indeed induced by ELS in APPswe/PS1dE9 mice, we measured plasma CORT levels under basal conditions, and at 30 and 90 min after a forced swim stress. Basal plasma CORT levels were comparable between the groups (Fig. 4a). Thirty minutes after acute stress, ELS-APPswe/PS1dE9 mice had higher plasma CORT levels than Ctrl-APPswe/PS1dE9 mice, while ELS-WT mice had lower plasma CORT levels than Ctrl-WT mice (interaction effect: F(1,40) = 14.54, *p* < 0.001; post hoc test: ELS-WT vs. ELS-APPswe/PS1dE9, *p* < 0.001; Ctrl-APPswe/PS1dE9 vs. ELS-APPswe/PS1dE9, *p* < 0.001; Ctrl-WT vs. ELS-WT, *p* = 0.03).

Ninety minutes after stressor onset, plasma CORT levels in ELS-APPswe/PS1dE9 mice were still elevated when compared to Ctrl-APPswe/PS1dE9 mice (interaction effect: F(1,41) = 8.22, *p* = 0.01; post hoc Tukey test: Ctrl-WT vs. ELS-WT, *p* = 0.04; ELS-WT vs. ELS-APPswe/PS1dE9, *p* = 0.02). Determining the total area under the curve of the plasma CORT response from 0 to 90 min after stressor onset revealed that ELS-APPswe/PS1dE9 mice were exposed to elevated plasma CORT levels after stress, relative to Ctrl-APPswe/PS1dE9 mice, and relative to ELS-WT mice (interaction effect: F(1,40) = 22.67, *p* < 0.001; post hoc Tukey test: ELS-WT vs. ELS-APPswe/PS1dE9, *p* < 0.001, Ctrl-APPswe/PS1dE9 vs. ELS-APPswe/PS1dE9, *p* < 0.001; Fig. 4b). Plasma CORT levels (area under the curve) further correlated positively with the latency on trial 1.2 of the reversal learning test (*r* = 0.40, *n* = 44, *p* = 0.01), i.e., the trial in which cognitive flexibility is best reflected (Fig. 4c).

### Brief treatment with mifepristone reduces Aβ pathology and rescues spatial memory deficits

**Fig. 5:**
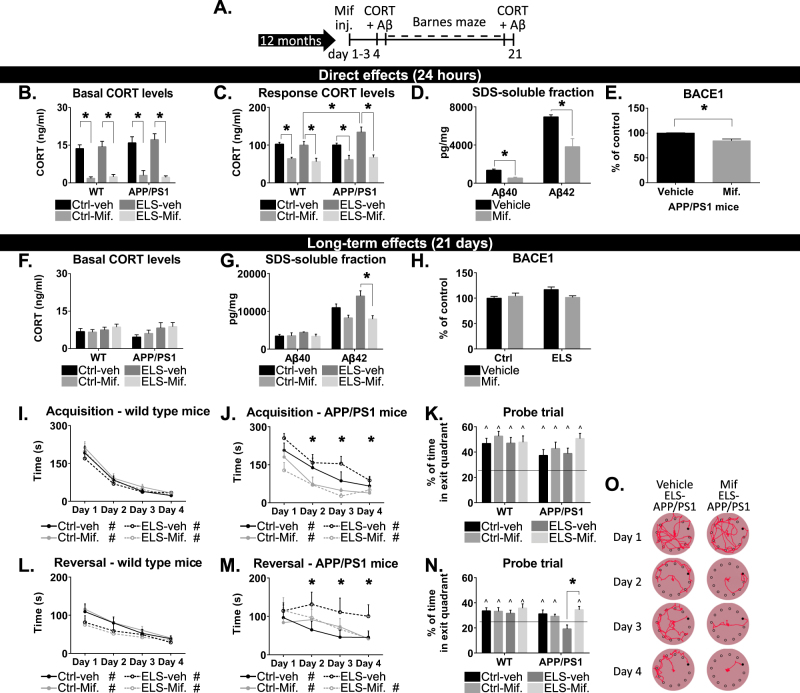
Three-day treatment with mifepristone lowers CORT levels, reduces Aβ pathology and improves spatial memory in ELS-APPswe/PS1dE9 mice. **a** Time schedule of the experiment. **b** Basal CORT levels are reduced 24 h after the last mifepristone treatment (*N* = 5 mice/group). **c** In response to an acute stressor, mifepristone treatment reduced plasma CORT levels (*N* = 8–10 mice/group). **d** Twenty-four hours after the last treatment, mifepristone treatment reduced hippocampal Aβ40 and Aβ42 levels (*N* = 4–5 mice/group). **e** BACE1 expression is reduced 24 h after the last mifepristone treatment. **f** Twenty-one days after the last treatment, no differences in basal CORT levels were observed. **g** Twenty-one days after the last treatment, there were no differences in the levels of Aβ40 between the groups. Aβ42 levels remained lower after mifepristone treatment, with significant post hoc tests between the ELS groups. **h** Twenty-one days after the mifepristone treatment, no differences are observed in the expression of BACE1. **i** In WT mice, mifepristone treatment had no effect on acquisition learning on the Barnes maze, and all mice learned to locate the exit hole. **j** In APPswe/PS1dE9 mice, mifepristone treatment resulted in a decreased time to locate the exit hole. **k** During the probe trial, all groups spend significantly more than 25% of the time in the exit quadrant. **l** In WT mice, mifepristone treatment had no effect on reversal learning. **m** In APPswe/PS1dE9 mice, mifepristone treatment resulted in a decreased time to locate the exit hole, specifically in ELS-APPswe/PS1dE9 mice. All mice treated with mifepristone learned to locate the exit hole; vehicle treated mice did not. **n** In the probe trial of the reversal learning, mifepristone treatment resulted in an increase in the time spend in the exit quadrant. Post hoc Tukey revealed a significant difference between ELS-APPswe/PS1dE9-veh and ELS-APPswe/PS1dE9-mifepristone mice. All experiments at 21 days used *N* = 7–13 mice/group. Mif. mifepristone. * indicates a significant post hoc Tukey test. # indicates a significant learning curve over the days. ^ indicates significant performance compared to chance level

To investigate whether blocking the GR, i.e., the receptor that becomes selectively occupied by CORT only during stress, could interfere with Aβ pathology and cognitive decline in ELS-APPswe/PS1dE9 mice, animals were treated for 3 days with mifepristone at 12 months of age (Fig. 5a). To validate the acute effects of mifepristone treatment on relevant parameters, APPswe/PS1dE9 mice were sacrificed 24 h after the last treatment. Mifepristone reduced both basal plasma CORT levels (treatment effect: F(1,31) = 111.46, *p* < 0.001; post hoc: Ctrl-WT-veh vs. Ctrl-WT-Mif, *p* < 0.001; ELS-WT-veh vs. ELS-WT-Mif, *p* < 0.001; Ctrl-APPswe/PS1dE9-veh vs. Ctrl-APPswe/PS1dE9-Mif, *p* < 0.001; ELS-APPswe/PS1dE9-veh vs. ELS-APPswe/PS1dE9-Mif, *p* < 0.001) and the elevation in plasma CORT levels after foot shock stress (F(1,56) = 53.53, *p* < 0.001; post hoc: Ctrl-WT-veh vs. Ctrl-WT-Mif, *p* < 0.01; ELS-WT-veh vs. ELS-WT-Mif, *p* = 0.001; Ctrl-APPswe/PS1dE9-veh vs. Ctrl-APPswe/PS1dE9-Mif, *p* = 0.05; ELS-APPswe/PS1dE9-veh vs. ELS-APPswe/PS1dE9-Mif, *p* < 0.001; ELS-WT-veh vs. ELS-APPswe/PS1dE9-veh, *p* = 0.04; Fig. 5b, c). Twenty-four hours after mifepristone treatment, hippocampal levels of Aβ40 and of Aβ42 were reduced by 60% and 45%, respectively (Aβ40: *t*(4) = 4.81, *p* = 0.01; Aβ42: *t*(4) = 3.52, *p* = 0.02; Fig. 5d), while BACE1 levels in the hippocampus were also reduced (*t*(6) = 3.12, *p* = 0.02; Fig. 5e).

Twenty-one days after mifepristone treatment, no differences were present in basal plasma CORT levels between any of the groups (*t*(15) = 0.89, *p* = 0.39; Fig. 5f). However, the levels of Aβ42 remained significantly reduced in ELS mice that had been treated with mifepristone (F(1,18) = 15.08, *p* < 0.001; post hoc: ELS-mifepristone vs. ELS-vehicle, *p* < 0.001), while Aβ40 levels were comparable between vehicle and mifepristone-treated APPswe/PS1dE9 mice (treatment effect: F(1,20) = 0.73, *p* = 0.40; condition effect: F(1,20) = 0.56, *p* = 0.46; Fig. 5g). No effects of mifepristone on BACE1 were present at twenty-one days after treatment (condition effect: F(1,20) = 1.62, *p* = 0.22; treatment effect: F(1,20) = 2.57, *p* = 0.12; interaction effect: F(1,20) = 4.32, *p* = 0.051; Fig. 5h).

During acquisition learning, APPswe/PS1dE9 mice were slower in acquisition learning on the Barnes maze (F(1,32) = 14.94, *p* < 0.001; Fig. 5i, j). Notably, mifepristone improved cognitive performance in both ELS-APPswe/PS1dE9 and Ctrl-APPswe/PS1dE9 mice (acquisition learning: treatment effect, F(1,25) = 11.78 and *p* < 0.001; probe trial treatment effect, F(1,58) = 4.66 and *p* = 0.035; no post hoc Tukey differences detected; Fig. 5j, k), while the treatment did not affect performance in WT mice (F(1,38) = 1.05, *p* = 0.31; Fig. 5i). Upon reversal learning, mifepristone also rescued the cognitive impairments specifically in ELS-APPswe/PS1dE9 mice, but did not alter performance in WT mice (WT: F(1,38) = 0.21, *p* = 0.65; APPswe/PS1dE9: F(1,60) = 4.80, *p* = 0.03; post hoc Tukey: *p* = 0.01; Fig. 5l–o).

## Discussion

We report that exposure of genetically predisposed mice to ELS from PND 2 to 9 exacerbates the development of amyloid pathology and accelerates cognitive decline, notably in close association with enhanced HPA axis activity. Blocking GRs with the GR antagonist mifepristone for only 3 days normalised the elevated plasma CORT levels, reduced Aβ and BACE1 levels, and rescued the cognitive impairments in 12-month-old ELS-APPswe/PS1dE9 mice.

Acquisition learning in the Barnes maze was clearly impaired in 12-month-old APPswe/PS1dE9 mice, consistent with earlier observations^33^. Only the ELS-APPswe/PS1dE9 mice, however, were hampered in their reversal learning/cognitive flexibility, a domain also specifically affected in early stages of AD^34^. Notably, this reduction in cognitive flexibility was not observed in ELS-WT mice. Since the ELS-APPswe/PS1dE9 mice also showed enhanced Aβ pathology, this suggests these behavioural deficits are mediated by elevations in brain amyloid levels. In line with this, the Aβ42 levels and Barnes maze performance were strongly correlated, both during acquisition and retrieval. ELS may thus specifically promote increases in Aβ42 levels, likely via changes in BACE1, which, over time, could impair cognitive flexibility. Clearly, age is a relevant factor too as increases in hippocampal Aβ42 levels and plaque load were already found after ELS in 6-month-old APPswe/PS1dE9 mice, but reduced cognitive flexibility was not apparent yet at that age. Possibly, the absolute Aβ levels may need to be further increased before they affect cognition.

Age-related cognitive decline has previously been associated with age-related increases in HPA axis activity^35,36^. In addition, elevated basal levels of circulating cortisol in the early disease stages^37–39^, as well as the failure to suppress cortisol after the dexamethasone challenge^40–42^, indicates that HPA axis activity is altered in AD patients. Notwithstanding, HPA dysfunction does not seem to worsen further with disease progression in patients^43,44^, implying that early alterations in HPA axis, likely acting via glucocorticoids, contribute to the onset and subsequent acceleration of AD pathogenesis. When this occurs in the context of an ELS history, such changes may possibly be amplified with increasing age^45–47^. In agreement, stress-induced CORT levels were exclusively enhanced in the ELS-APPswe/PS1dE9 mice.

In addition, and in line with the hypothesis that changes in HPA axis activity contribute to the accelerated cognitive decline in ELS-APPswe/PS1dE9 mice, we could rescue the reduction in cognitive flexibility by a brief treatment with the GR antagonist mifepristone. This effect was, notably, most prominent in the ELS-APPswe/PS1dE9 mice. Moreover, ELS caused increases in both Aβ40 and Aβ42 levels at 6 and in Aβ42 levels at 12 months of age (Fig. 1a, b), while mifepristone treatment at 12 months of age strongly reduced both Aβ species (Fig. 5e, f) in the APPswe/PS1dE9 mice, again indicative of an amyloid-related mechanism. This is supported by earlier studies that used a much longer treatment period or a much higher dosage in other AD mouse models^12,48^. Because of its short treatment duration and relatively low dose, our current 3-day mifepristone treatment provides considerable practical advantages. The rescue effects we report here occur at an age at which the cognitive impairments and amyloid pathology have already manifested. Moreover, this short treatment regime not only rescued the cognitive and neuropathological phenotypes, but, importantly, these effects also persisted for at least 3 weeks.

An outstanding question remains how early stress and mifepristone treatment could affect amyloid pathology in the APPswe/PS1dE9 mice. ELS did not affect the levels of full-length APP, indicating that the changes in Aβ levels result either from altered processing of APP or from altered Aβ clearance. The rate-limiting enzyme involved in processing of APP to Aβ is BACE1, an enzyme that also contains glucocorticoid-binding sites^49^. Hence, increases in BACE1 expression following elevated CORT levels could, in time, lead to a higher and prolonged Aβ production and an earlier plaque accumulation. Indeed, BACE1 expression was significantly enhanced following ELS in APPswe/PS1dE9 mice, and mifepristone treatment rapidly reduced BACE1 expression, indicative of a common mechanism via which ELS and mifepristone affect Aβ levels.

Although the exact cellular mechanisms through which mifepristone reduces Aβ levels and prevents cognitive decline remain to be further investigated, blocking GC actions is an attractive option for possible future therapeutic interventions. So far, a small clinical trial in AD patients and old macaques monkeys reported improvements in cognition after mifepristone treatment^26,50^, although the short time window and small sample size warrant caution in interpreting these results. Furthermore, AD patients with the highest baseline cortisol levels benefited most from a mifepristone intervention and showed persistent memory improvements up to 8 weeks after discontinuation of the treatment^26^. This is in line with our findings that after 3 weeks, Aβ42 levels were still strongly reduced in the ELS-APPswe/PS1dE9 mice treated for 3 days with mifepristone. While this highlights an interesting translational potential of the drug and suggests it can ‘re-set’ earlier established changes, it also calls for further study of the mechanisms underlying these intriguing lasting effects already induced after such a short treatment with mifepristone.

Taken together, exposure to stress early in life, likely via the associated alterations in HPA axis activity, can exacerbate amyloid pathology at a later age. ELS upregulates Aβ levels and BACE1 expression, which may underlie cognitive deficits such as reversal learning, and may thus be a risk factor for AD in vulnerable individuals. Given the current rescue effects on both Aβ levels and cognitive flexibility in middle-aged mice already after a short mifepristone treatment, interventions with this classic drug, or other compounds targeting the HPA axis, may provide potential therapeutic benefits for AD, even at ages when symptoms have already manifested.

## Electronic supplementary material

Supplementary table 1
