# Supplementary material for: Targeting glucocorticoid receptors prevents the effects of early life stress on amyloid pathology and cognitive performance in APP/PS1 mice
Source: Transl Psychiatry. 2018 Mar 1;8:53. doi: 10.1038/s41398-018-0101-2 (PMC5830444; doi:10.1038/s41398-018-0101-2)
Supplement: Supplementary file 1 — Supplementary table 1 [file 41398_2018_101_MOESM1_ESM.pdf]

Supplementary table 1. Effects of chronic ELS exposure on pups (PND 9), 6 and 12 month old mice.

		Ctrl		ELS	
		**Light phase**	**Dark phase**	**Light phase**	**Dark phase**
**Maternal care (PND 2—9)**	Duration nursing (s)	2667 ± 46 *(8)*	484 ± 45 *(8)*	2323 ± 50 *(10)***	458 ± 52 *(8)*
	Duration off pups (s)	182 ± 40 *(8)*	561 ± 43 *(8)*	454 ± 54 *(10)***	469 ± 54 *(8)*
	Number of exits	0.5 ± 0.2 *(8)*	2.4 ± 0.4 *(8)*	2.4 ± 0.4 *(10)***	4.7 ± 0.9 *(8)***
	# pup(s) out of nest	0.0 ± 0.0 *(8)*	0.0 ± 0.0 *(8)*	4.8 ± 1.3 *(10)***	3.4 ± 0.8 *(8)***
		**Ctrl**		**ELS**	
**Pups**	Body weight gain PND 2-9 (g)	3.47 ± 0.11 *(24)*		2.60 ± 0.09 *(27)***	
	Body weight PND 21 (g)	8.80 ± 0.23 *(24)*		8.42 ± 0.23 *(27)*	
		**Ctrl - WT**	**Ctrl – APP/PS1**	**ELS – WT**	**ELS – APP/PS1**
**6 months**	Body weight (g)	29.4 ± 0.89 *(12)*	29.7 ± 0.34 *(10)*	28.9 ± 0.55 *(11)*	29.7 ± 0.38 *(14)*
	Thymus weight (% of BW)	0.159 ± 0.007 *(8)*	0.154 ± 0.003 *(6)*	0.147 ± 0.007 *(10)*	0.148 ± 0.004 *(6)*
	Adrenal gland weight (% of BW)	0.0062 ± 0.001 *(8)*	0.0076 ± 0.001 *(7)*	0.0096 ± 0.001 *(11)*[a]	0.0085 ± 0.001 *(7)*[a]
**12 months**	Body weight (g)	39.43 ± 1.50 *(13)*	39.13 ± *(9)*	38.65 ± 1.13 *(17)*	42.43 ± 1.75 *(9)*
	Thymus weight (% of BW)	0.109 ± 0.005 *(13)*	0.092 ± 0.004 *(7)*[b]	0.090 ± 0.006 *(16)* [b]	0.107 ± 0.010 *(9)* [b]
	Adrenal gland weight (% of BW)	0.013 ± 0.001 *(14)*	0.013 ± 0.001 *(9)*	0.011 ± 0.0008 *(15)*[a]	0.011 ± 0.0007 *(7)*[a]

Data expressed as mean ± S.E.M (*n*).

* $p<0.05$, t-test compared to Ctrl mice

[a] $p<0.05$, two-way ANOVA, main effect for condition.

[b] $p<0.05$, two-way ANOVA, interaction effect.
